# Why Does Mucoadhesion Matter? Mucoadhesive Drug Delivery Systems with Antifungal Activity in the Local Treatment of Oral Cavity Candidiasis

**DOI:** 10.3390/ma19010033

**Published:** 2025-12-21

**Authors:** Katarzyna Olechno, Julia Higuchi, Katarzyna Winnicka

**Affiliations:** 1 Department of Pharmaceutical Technology, Medical University of Bialystok, Mickiewicza 2c, 15-222 Bialystok, Poland; katarzyna.winnicka@umb.edu.pl; 2 Laboratory of Nanostructures, Institute of High Pressure Physics, Polish Academy of Sciences, Sokołowska 29/37, 01-424 Warsaw, Poland; j.higuchi@labnano.pl

**Keywords:** mucoadhesive drug carriers, local drug delivery systems, mucoadhesive polymers, mucoadhesion, antifungal therapy

## Abstract

Disorders in the oral cavity caused by pathogenic fungi pose a significant clinical challenge, particularly in immunocompromised patients, as well as those undergoing oncological therapy or antibiotic treatment. A practical therapeutic approach involves the topical application of mucoadhesive drug dosage forms. However, only a limited number of such preparations are available on the pharmaceutical market. Mucoadhesive systems are especially advantageous, as they ensure prolonged retention and adequate concentrations of the active substances at the site of infection. Localized drug delivery enhances therapeutic efficacy compared to systemic administration, enabling lower drug doses, and consequently reducing the risk of side effects. Moreover, many fungal conditions require long-term treatment, which may be optimized by the use of mucoadhesive systems, improving patient compliance. Considering the issue of limited adhesion of conventional drug dosage forms and the moist environment in the oral cavity, providing optimal mucoadhesive properties is a key aspect. This article presents a comprehensive overview of the significance of treating oral candidiasis using mucoadhesive drug dosage forms, the mechanisms and advantages of mucoadhesion (including relevant polymers), and, most importantly, recent scientific reports on the development and quality assessment of mucoadhesive drug delivery systems for the management of oral fungal diseases.

## 1. Introduction

### Oral Cavity as the Area of Drug Application

The oral cavity is a crucial and increasingly popular site for drug delivery, particularly for medications targeting mucosal membranes. The oral mucosa, with its complex structure and high permeability, provides a unique environment for the local application ion of drugs. Medications delivered in this manner can effectively treat various conditions, including microbial infections (such as candidiasis, gingivitis, periodontitis), as well as ulcers, inflammation, and pain. The oral mucosa consists of a multilayered squamous epithelium supported by an underlying lamina propria of connective tissue. The epithelium may be keratinized, as observed in masticatory regions such as the hard palate and gums, or non-keratinized, as in lining areas including the cheeks, lips, and the floor of the mouth. The lamina propria is contains a dense network of capillaries, fibroblasts, immune cells, and extracellular matrix components that provide structural support and facilitate immune responses. In certain regions, a submucosal layer is also present, containing minor salivary glands, adipose tissue, and neurovascular elements that play a crucial role in the absorption and delivery of drugs [1,2,3].

It should be emphasized that the oral cavity represents a highly dynamic and challenging environment due to continuous saliva flow, enzymatic activity, chewing, and tongue movements [1]. These conditions often cause premature detachment of the drug formulation’s preparation, resulting in a short residence time. In addition, the oral cavity inherently provides a limited area available for drug absorption. Most conventional therapies for oral diseases, which primarily include mouth rinses, suspensions, gels, or systemic medications, have several drawbacks, such as short retention time in the oral cavity caused by saliva flow and mechanical removal by the tongue or food. Such treatments also exhibit poor drug retention at the target site, resulting in subtherapeutic levels, and require frequent dosing, which negatively affects patient compliance. Moreover, systemic side effects may occur when drugs are administered orally or intravenously to treat conditions localized in the oral cavity. These limitations highlight the need for site-specific, sustained, and patient-friendly drug delivery systems that can withstand the dynamic environment of the oral cavity. Mucoadhesive, polymer-based drug delivery systems offer several advantages over conventional formulations, including prolonged contact time with mucosal tissues, which enhances local accumulation and absorption. These systems also provide extended therapeutic effects and allow for the optimization of pharmacotherapy through the controlled release of therapeutic agents, as well as targeted delivery to specific tissues. By forming a protective barrier, they also shield the active substance from hydrolytic or enzymatic degradation, while masking unpleasant taste or smell. The mucoadhesive systems enhance dosing accuracy, often enabling dose reduction and thereby minimizing the risk of adverse effects. In addition, they reduce the frequency of drug administration, thereby increasing patient adherence to the treatment. To optimize mucosal drug delivery, several strategies are employed, including the development of mucoadhesive films, tablets, patches, and gels, all designed to adhere to the mucosal surface and provide sustained, localized drug release through the use of suitable polymers and their tailored modifications [1,4,5,6].

## 2. Methodology

A comprehensive literature search was conducted to identify scientific publications on mucoadhesive antifungal drug delivery systems designed for oral mucosal application in the treatment oral candidiasis. The search included original research articles and review papers indexed in PubMed, Scopus, Web of Science, and Embase, covering studies published from 2020 to 2025. Keywords and search terms used included: mucoadhesive, bioadhesive, oral drug delivery, buccal drug delivery, oromucosal, antifungal, oral candidiasis, mucoadhesive drug carriers, mucoadhesive polymers, mucoadhesion theory, mucoadhesion, buccal, and antifungal therapy. The identification process consisted of a two-step screening method. First, titles and abstracts were examined for initial relevance. Then, full-text articles deemed potentially suitable were retrieved and assessed based on predetermined inclusion criteria. Eligible studies included those describing mucoadhesive dosage forms for oral use in treating oral candidiasis, such as buccal tablets, mucoadhesive films, gels, patches, or nanocarriers, and those presenting aspects such as formulation design, characterization, performance testing, antifungal activity, therapeutic potential, or clinical relevance. Publications discussing mechanisms and theories of mucoadhesion, analytical methods for evaluating mucoadhesive properties, and etiological or clinical aspects of oral candidiasis were also included to establish a strong theoretical foundation. All identified records were thoroughly screened.

## 3. Fungal Oral Diseases

Oral diseases associated with fungal infections represent a significant therapeutic challenge with implications for both oral and systemic health. A healthy oral microbiome is characterized by a balanced microbial composition that prevents the overgrowth of pathogenic microorganisms through competitive interactions, immune modulation, and the production of antimicrobial agents. Oral candidiasis emerges when local or systemic factors disrupt the physiological balance, leading to dysbiosis [7,8,9]. Major risk factors include broad-spectrum antibiotic use, inhaled corticosteroid therapy, and immunosuppression, such as chemotherapy, radiotherapy, bone marrow transplantation, or AIDS. Additional risks comprise diabetes, nutritional deficiencies (such as iron, folate, and vitamin B_12_), salivary gland hypofunction (such as in Sjögren’s syndrome or post-radiotherapy), poor oral hygiene, high sugar intake, dentures use, smoking, or xerostomia. Furthermore, age is a predisposing factor for oral candidiasis, especially in pediatric and geriatric populations. Although often asymptomatic in healthy individuals, the fungal colonization may cause considerable morbidity in patients with compromised immune systems or underlying health problems. When left untreated, infections may result in discomfort, pain, and complications such as dysphagia, systemic dissemination, and impaired quality of life. Prompt and appropriate antifungal therapy is essential to prevent chronic infection, the emergence of antifungal resistance, and the spread of disease to other mucosal or systemic sites. Early intervention therefore plays a vital role in maintaining oral microbiota balance. Oral candidiasis usually manifests as white, creamy lesions occurring on the mucous membranes of the mouth, and, in more severe cases, it may extend to the throat or esophagus. The predominant causative agents of oral candidiasis are species of the genus *Candida*, especially *C. albicans*, which accounts for approximately 70–90% of all cases. Other species, such as *C. glabrata*, *C. tropicalis*, *C. krusei*, *C. parapsilosis*, and *C. dubliniensis*, may also be implicated, particularly in patients with recurrent infections or those suffering from those underlying systemic diseases. Table 1 presents the classification of oral candidiasis [8,10,11,12,13,14,15,16].

## 4. Bioadhesion Phenomenon

Bioadhesion is defined as the bonding between two different biological materials or the adhesion of a biological material, most often cells or their secretions (e.g., mucus) to the surface of a polymeric layer. When referring specifically to interactions between materials and the mucosal membrane, the term *mucoadhesion* is used, representing a distinct subtype of bioadhesion. Mucoadhesion has garnered considerable interest in recent years as a strategy to enhance drug delivery and therapeutic efficacy, particularly through the mucosal routs such as oral, nasal, or vaginal membranes [26,27,28]. Mucus is a highly viscous hydrogel composed mainly of water (about 95%), lipids, inorganic salts, and mucin. The mucins constitute a group of glycoproteins consisting of a protein core, composed mainly of serine, threonine, proline, and side chains of polysaccharides formed from monosaccharides, such as N-acetylglucosamine, D-galactose, N-acetylgalactosamine, and sialic acid, which are cross-linked mainly via disulfide bonds between cysteine residues. This creates a viscoelastic network that provides a protective barrier over mucosal surfaces. At physiological pH, mucus typically has a negative charge, which is attributed to the presence of sialic acid (pKa ≈ 2.6) and sulfated esters of polysaccharides in its composition. This property is highly important for mucoadhesion, as it facilitates retention and adhesion of drug dosage forms at the site of application [28,29,30,31,32,33].

The mucoadhesive process of forming bioadhesive bonds between the polymer and mucin involves three consecutive stages: wetting and swelling of the polymer, mutual penetration with entanglement of the polymer and mucin chains, and the formation of chemical bonds between the chains. Chemical interactions between polymer and mucin chains typically occur through hydrogen and hydrophobic interactions or Van der Waals forces [28,30]. Additionally, stronger bonds can be established through ionic or covalent interactions. Hydrophilic mucoadhesive polymers tend to create stronger hydrogen bonds because of their affinity for water molecules in mucus. It is important to note that the strength of polymer adhesion to the mucosal membrane is influenced by numerous factors, including the polymer’s molecular weight (with adhesion typically increasing as molecular weight rises), chain flexibility enabling penetration into the mucin layer, hydrogen-bonding capacity, crosslinking density, electrical charge, swelling behavior, and polymer concentration. Insufficient polymer concentration results in polymer–mucin interactions, while excessively high concentrations may limit wetting and reduce adhesiveness. It is crucial to consider that saliva, containing enzymes, mucins, and electrolytes, can influence the stability and retention of mucoadhesive materials. Additionally, salivary flow can physically remove the formulation, while the pH of the oral environment can modify the solubility or ionization state of certain polymers, affecting their adhesive behavior. For instance, acidic or basic conditions might weaken the interaction between the polymer and the mucosal surface by altering charge distribution or molecular conformation [28,30,34,35,36,37,38].

Furthermore, it is important to consider the effects of temperature, pH, and ionic strength, as these factors significantly influence the physicochemical properties of mucoadhesive polymers and their interactions with the tissue. Temperature affects polymer hydration, chain mobility, and potential gel formation, thereby influencing both the extent and rate of polymer–mucin interpenetration as well as the formation, strength, and stability of non-covalent interactions, including hydrogen bonding and van der Waals forces. pH determines the ionization states of functional groups in both mucoadhesive polymers and mucins, regulating electrostatic forces that are vital for electronic and adsorption interactions. This is especially relevant for pH-responsive systems based on chitosan, alginate, pectin, and thiolated polymers, the mucoadhesive abilities of which vary significantly with the protonation or deprotonation of functional groups. Ionic strength further modulates mucoadhesion by altering polymer chain conformation, screening mucin surface charges, and alerting the effective crosslinking density of the hydrated polymer matrix. Variations in ionic strength can thus enhance or attenuate wetting behavior, hydrogen bonding capacity, and electrostatic attraction at the polymer–mucosa interface [35,36,37].

### 4.1. Mucoadhesion Theories and Analysis: Techniques for the Assessment of Mucoadhesion in Oral Drug Dosage Forms

Given the complexity and diversity of phenomena related to mucoadhesion, several theories have been proposed to explain the interactions. The wetting theory posits that adhesion arises from the intimate molecular contact between two surfaces and the surface forces that develop at their interface. Accordingly, the adhesive affinity of a liquid or semi-liquid formulation toward the tissue depends on intermolecular interactions and the surface tension between the formulation and the substrate. In practical terms, this means that a mucoadhesive material must first adequately wet the tissue to spread and establish intimate contact. The greater the wettability and hydrophilicity of the polymer, the more favorable the adhesion [28,37]. Wettability can be quantitatively assessed using the contact angle: lower angles indicate better spreading and, consequently, a higher mucoadhesive potential. Wetting theory therefore primarily applies to liquid, low-viscosity, and semi-solid systems. The diffusion theory of mucoadhesion suggests that mucoadhesive polymers adhere to the mucosal surface by penetrating polymer chains into the mucus layer, which leads to the formation of adhesive bonds, with the bond strength increasing as the penetration of the polymer chains into the tissue deepens. The extent of chain entanglement and diffusion depends on the molecular weight of the polymer and the interaction between the polymer and mucus. Structural similarity between the polymer and mucin ensures mutual solubility, facilitating the formation of sufficiently strong bonds [36,37]

According to the adsorption theory, mucoadhesion arises from intermolecular interactions that develop once intimate contact is established between the polymer and the mucosal surface. Such interactions may involve primary bonds (ionic or covalent), although strong, permanent linkages are generally undesirable in mucoadhesive systems. Predominantly, secondary interaction, including hydrogen bonding, van der Waals forces, hydrophobic interactions, and electrostatic attraction, are responsible for adhesion. The electrostatic theory assumes that the polymer and mucin glycoproteins carry different electrical charges, e.g., negatively charged mucin interacts with positively charged mucoadhesive materials, such as chitosan. As a result, the electrons flow and form a double layer of electrical charge. The strength of adhesion depends on the ionic strength of the environment and the charge density of the mucosal polymer [28,37].

The fracture theory explains mucoadhesion in terms of the forces required to separate two surfaces once an adhesive joint has been formed. Accordingly, mucoadhesion is strengthened when the adhesive interface can resist external forces, such as mechanical stress, shear, or mucus flow, that attempt to pull the polymer away from the tissue. The detachment force depends on the mechanical properties of the adhesive joint, including its elasticity, cohesiveness, and resistance to fracture. It is generally observed that the detachment force increases with polymer chain length, due to enhanced chain mobility and interfacial entanglement with mucin. In contrast, a higher degree of crosslinking reduces chain flexibility and interpenetration, leading to lower adhesive strength [28,36,37].

One of the fundamental steps in the development of a mucoadhesive drug delivery system is evaluating the adhesive strength between the formulation and the mucosal membrane. Most quantitative methods described in the literature involve measuring the force required to detach the mucoadhesive formulation from a model of mucosal surface [39,40]. These tests evaluate the strength of the adhesive bonds formed between the polymer and the mucosa, providing essential data for predicting formulation’s performance under in vivo conditions. Depending on the direction of the applied detachment force, mucoadhesion studies are generally classified into tensile, shear, or peel tests [39,41]. Each method provides insights into the nature and robustness of adhesive interactions under various mechanical stresses, which is crucial for optimizing the properties of the formulation. However, mucoadhesion research relies on a wide variety of experimental methods and analytical techniques. No standardized apparatus or universally accepted protocol exists for assessing mucoadhesive performance. As a result, multiple in vitro and ex vivo tests are typically required to characterize mucoadhesion, yet these methods differ substantially in their substrates, measurement principles, environmental conditions, and data interpretation. This lack of uniformity and methodological consistency hinders direct comparison of results across studies and complicates the establishment of robust structure–adhesion relationships essential for formulation development. Table 2 provides a brief overview of the most commonly used in vitro methods for evaluating mucoadhesion.

**Table 2:** Short characteristics of commonly used methods for mucoadhesion evaluation [35,36,39,40,41].

Test Method	Characteristic
**Tensile test** (Figure 1)	Measurement of the maximum detachment force (Fmax)and the work of adhesion (Wad) required to detach a mucoadhesive material from a mucosal surface (or its substitute, e.g., gelatin layer) under controlled shear stress conditions. Quantify the strength of the adhesive bond formed between the formulation and mucus. Based on the fracture theory. Before measurement, the formulation is applied to a pre-moistened mucosal surface or its substitute, followed by the application of a defined compressive force to ensure close contact between the layers. The relationship between the detachment force and time or displacement is recorded. The area under the force–time or force–displacement curve obtained during the detachment process, expressed in units of work, corresponds to the work of mucoadhesion (Wad). Usually performed using a texture analyzer, which pulls the sample vertically at a constant rate, recording force over time or displacement. The detachment curve provides peak detachment force Fmax and area under the curve (Wad), reflecting both strength and duration of adhesive interactions. Particularly useful in comparative formulation development, screening polymer blends, and assessing the influence of polymer properties (e.g., crosslinking density, hydration capacity). Advantages: provides quantitative, reproducible measurements of mucoadhesive force; sensitive to formulation parameters such as swelling, polymer concentration, functionalization (e.g., thiolation), and surface interactions; applicable to solid, semi-solid, and gel dosage forms; allows precise control of test variables (force, speed, contact time, preload). Limitations: measures only normal (vertical) detachment, whereas in vivo mucoadhesion often involves shear and combined stresses; contact time and pre-load selection significantly affect outcomes; lack of standardization makes inter-study comparison difficult.
**Contact angle measurement** (Figure 2)	Measurement of the wettability of a mucoadhesive material by quantifying the angle formed between a liquid droplet and the solid surface of the formulation. The parameter reflects the ability of a material to spread over the moist mucosal surface, thereby influencing its adhesive performance. Based on wetting theory, in which the contact angle (θ) describes the balance of interfacial tensions at the liquid–solid–air interface according to Young’s equation A small droplet of liquid is placed on the surface of the mucoadhesive material, and the angle formed at the liquid–solid interface is measured using a contact angle goniometer. Low contact angle (<90°): the droplet spreads readily→high wettability→greater potential for mucoadhesion. High contact angle (>90°): limited spreading→poor wettability→reduced adhesive potential. Advantages: Simple, fast, and nondestructive method; allows precise quantification of surface wettability; provide insight into early mucoadhesion stages (wetting and spreading). Limitations: measures only the initial wetting behavior, not the full mucoadhesion process; surface roughness and heterogeneity may distort angle measurements; difficult to compare results across studies due to lack of methodological standardization.
**Swelling test**	Used to assess the hydration behavior, liquid uptake capacity, and physical changes in mucoadhesive dosage forms upon exposure to aqueous environments mimicking mucosal conditions. The method provides indirect insight into the mucoadhesive properties of the material, as swelling plays a crucial role in determining the extent of interactions between the polymer and mucus. Advantages: provides quantitative hydration and swelling kinetics; helps predict polymer relaxation and gel formation behavior relevant to mucoadhesion. Limitations: indirect measure of mucoadhesion—swelling alone does not guarantee adhesive Typically, a pre-weighed mucoadhesive sample is immersed in a simulated mucus fluid (e.g., phosphate-buffered saline or artificial saliva) maintained at body temperature (37 ± 0.5 °C). The sample is removed at set time intervals, then gently dried to remove excess moisture, and reweighed. The swelling degree is being calculated using the formula: *Swelling Index (SI) = (W_t_ − W_0_)/W_0_* W_t_—the weight of the swollen sample at time t; W_0_—the initial dry weight.
**Rheological analysis**	Used to evaluate mucoadhesive interactions between polymers and mucin involving quantifying changes in viscosity and viscoelastic properties upon mixing. Enables an indirect prediction of in vivo performance by characterizing the mechanical behavior, structural integrity, and interaction strength of mucoadhesive formulations. An increase in viscosity, storage modulus (G′), loss modulus (G″), or complex modulus (G*) typically indicates synergistic interactions associated with polymer–mucin network formation. The interactions are commonly quantified using a rheological interaction parameter (Δη or Δη*), which compares the measured properties of the mixture with those of the individual components. The measurements may include steady–shear testing to characterize flow behavior across varying shear rates, as well as oscillatory rheology to assess elastic and viscous contributions to the system. Advantages: allows assessment of both viscous and elastic contributions to mucoadhesion; may mimic dynamic physiological conditions (changing shear rates, oscillation frequencies). Limitations: indirect measure (not assess adhesion to actual mucosal tissue).

**Figure 1:**
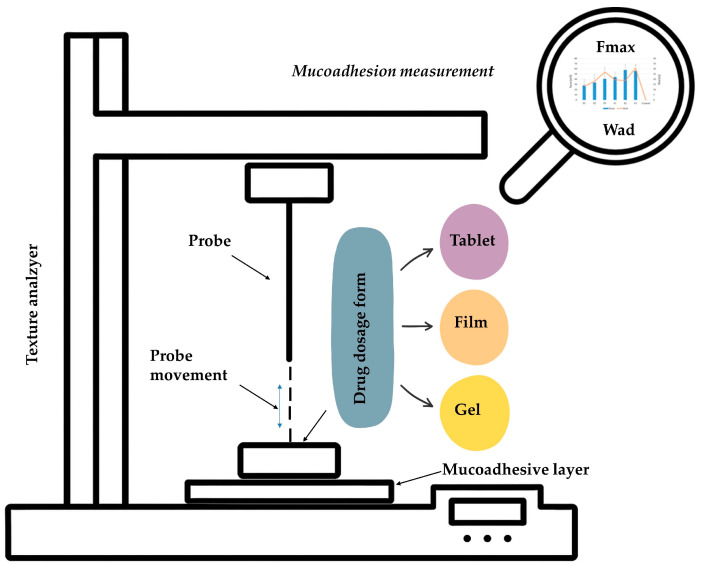
Schematic illustration of the texture analyzer in terms of mucoadhesion measurement.

**Figure 2:**
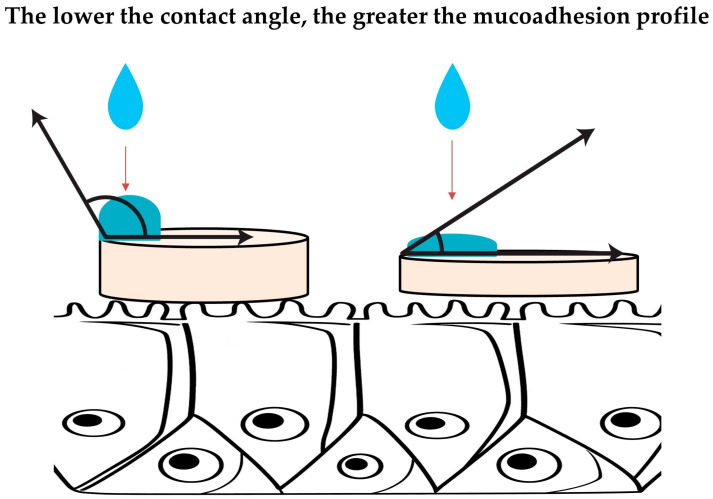
Schematic illustration of contact angle measurement.

### 4.2. Polymers Utilized in the Design of Mucoadhesive Drug Dosage Forms

Mucoadhesive polymers, which adhere to mucosal surfaces, have become an essential component of modern drug delivery systems because they provide sustained drug release at the application site by prolonging contact with the mucosal membrane. By adhering directly to the mucosal surface, the drug is delivered precisely to the site of action, thereby reducing systemic distribution and minimizing side effects. Localized drug concentration within the mucosal tissue reduces the need for higher doses, improves the bioavailability of the active ingredient, thereby increasing treatment effectiveness while minimizing adverse effects associated with systemic administration. Mucoadhesive polymers remain attached to the mucosa, ensuring prolonged retention and more consistent drug release. Moreover, they form a protective layer over the oral mucosa, shielding the drug from being washed away by saliva and helping to reduce microbial proliferation [42,43,44,45].

It should be noted that the thiolation of polymers has attracted significant attention as a strategy for developing mucoadhesive formulations with improved mucoadhesive and mechanical properties, as well as enhanced drug retention and bioavailability at the targeted site. The introduction of thiol groups enables the formation of disulfide bonds with mucus glycoproteins (cysteine-rich areas). Chitosan, alginate, hyaluronic acid, gelatin, and xanthan gum are most commonly modified due to their biocompatibility and abundance of reactive sites [46].

Mucoadhesive polymers can be broadly classified into two categories: natural and synthetic [44], each possessing unique characteristics that make them suitable for drug-delivery applications (Table 3).

## 5. Mucoadhesive Drug Delivery Systems with Antifungal Activity for Oral Candidiasis: A Literature Overview

Despite the recognized therapeutic potential of mucoadhesive formulations, no dedicated monograph in current pharmacopoeias or industrial guidelines provides harmonized quality standards or manufacturing methods for mucoadhesive buccal tablets or films. There is also a lack of standardized protocols for assessing the mucoadhesive properties of these formulations. In the absence of detailed pharmacopoeial monographs for buccal tablets, the general requirements for uncoated tablets are applied [58,59]. To date, the only widely approved mucoadhesive product for the management of oral candidiasis on the pharmaceutical market is the mucoadhesive buccal tablet of miconazole, marketed as Loramyc^®^ in Europe and Oravig^™^ in the United States [60,61]. Additionally, topical antifungal formulations, such as Daktarin^®^ Oral Gel (miconazole) and Mycelex^®^ Oral Gel (clotrimazole) intended to be applied to the oral mucosa are also available [62,63].

The limited regulatory framework, coupled with the small number of authorized products, highlights a significant medical and technological need in this field, in the light of the benefits offered by mucoadhesive drug dosage forms. These advantages have motivated considerable research and innovation toward designing and evaluating novel mucoadhesive delivery systems for antifungal agents. Recent reports show that developing mucoadhesive drug formulations remains a prominent and widely discussed area in the management of oral candidiasis. Various mucoadhesive systems, such as films, tablets, patches, or gels, have been developed using advanced technologies [6].

It is important to note that integrating micro- and nanotechnology is critical in advancing mucoadhesive drug delivery systems. The development of carriers at micro- and nanoscale dimensions significantly increases the surface area available for interaction with mucosal tissues, thereby enhancing adhesion and extending residence time at the target site. Moreover, these carriers can be engineered for controlled and sustained drug release, maximizing therapeutic effectiveness while reducing systemic side effects. Their small size and specific structural features may facilitate deeper penetration into mucosal layers, increasing drug bioavailability and enabling more precise delivery to the affected tissues. Incorporating such technologies into the designed mucoadhesive drug delivery systems offers a promising approach to overcoming mucosal drug delivery barriers related to solubility and permeability, ultimately leading to more effective and patient-friendly treatments. Of particular importance is that higher permeation may enhance therapeutic effect, allowing for dose reduction, while ensuring an optimal therapeutic concentration of active substance at the targeted site. Additionally, micro- and nanocarriers can shield sensitive drugs from enzymatic degradation within the oral cavity and improve their solubility and stability, addressing issues of low and variable bioavailability that frequently limit their clinical use [64,65].

The scarcity of drugs, rising resistance, and the predominance of systemic treatments, often associated with side effects and drug interactions, pose significant challenges. Consequently, there is a growing need to develop new antifungal agents, improve existing ones, and explore alternative technological approaches and drug delivery strategies that provide safe and effective therapy while minimizing adverse effects. Moreover, due to increasing drug resistance among pathogens, there is ongoing interest in the other biological activities of existing active substances and in repositioning drugs by altering their routes of administration [66].

An overview of recent advances (within the past five years) in the design and development of antifungal mucoadhesive drug delivery systems is presented in Table 4 and discussed further below. It is important to emphasize that several of the agents incorporated into the reviewed mucoadhesive systems (α-mangostin [67], probiotic extracts [68], lectins [69], atorvastatin [70,71], and triamcinolone acetonide [72]) are not conventional antifungal drugs, nor are they currently recommended in clinical guidelines for the treatment of oral candidiasis. These substances represent experimental or repurposed therapeutic approaches, and their use remains off-label. However, the antifungal potential of probiotics and statins is supported by growing evidence in the literature [73,74,75,76,77,78,79], with multiple studies demonstrating their activity against pathogenic fungi. In contrast, α-mangostin, lectins, and triamcinolone acetonide represent more innovative or exploratory candidates, whose antifungal effects are still under investigation.

### 5.1. Solid Drug Dosage Forms

**Figure 3:**
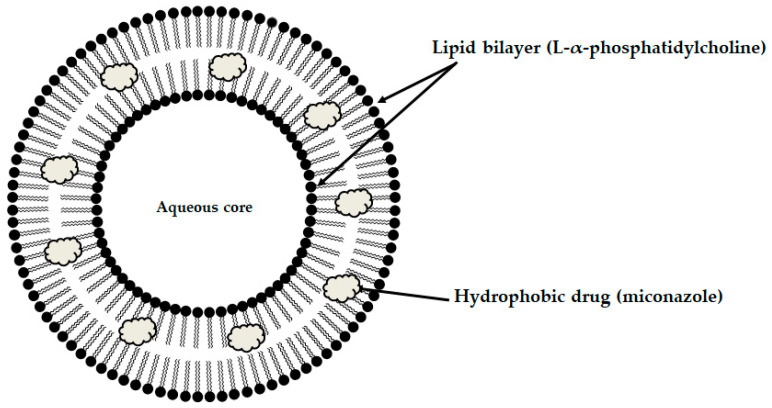
Schematic illustration of the designed liposomes.

**Figure 4:**
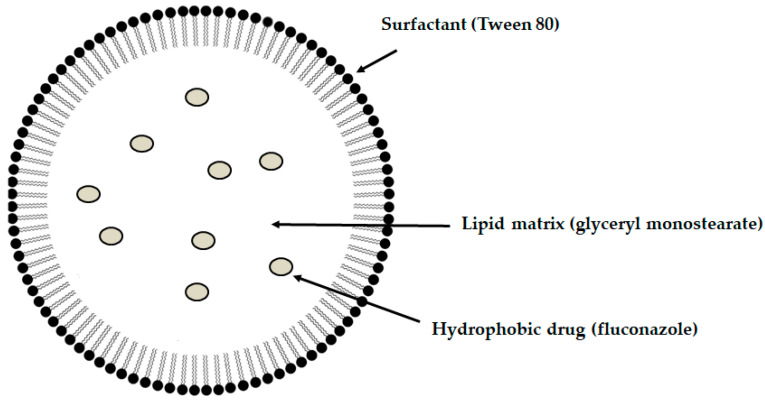
Schematic illustration of the designed solid lipid nanoparticle.

**Figure 5:**
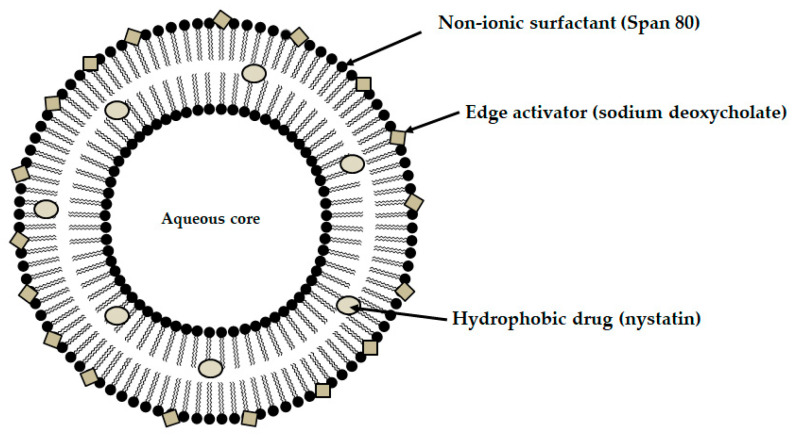
Schematic illustration of the designed spanlastic.

**Figure 6:**
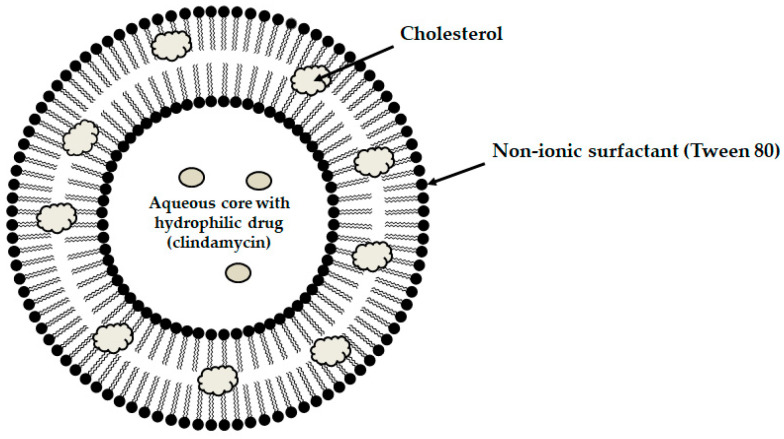
Schematic illustration of the structure of the designed niosome.

**Figure 7:**
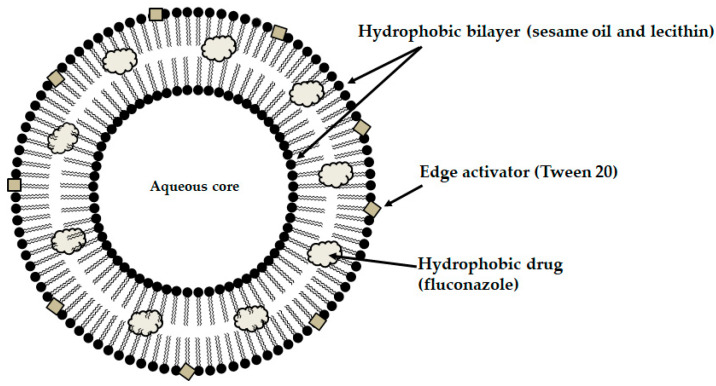
Schematic illustration of the designed nanotransferosome.

Among the mucoadhesive drug dosage forms designed for application to the oral mucosa, solid formulations are recognized as the foundation of this group of therapeutic systems. They offer the key advantages such as persistence, reproducibility, extended retention within the oral cavity, and ease of application [6]. Considerable attention has been directed toward the design of mucoadhesive films and tablets. In recent years, research into advanced mucoadhesive drug delivery systems has also emphasized the potential use of carriers such as liposomes (Figure 3) [82], solid lipid micro- or nanoparticles (Figure 4) [83], spanlastics (Figure 5) [94], niosomes (Figure 6) [97], or nanotransferosomes (Figure 7) [101].

Liposomes consist of a phospholipid bilayer surrounding an aqueous core, enabling the incorporation of both hydrophilic and hydrophobic compounds—hydrophilic agents are entrapped within the aqueous core, whereas lipophilic agents partition into the lipid bilayer, thereby facilitating targeted delivery to the intended site of action [104,105,106]. Niosomes and spanlastics are vesicular carriers mainly of non-ionic surfactants, offering advantages such as improved stability, biocompatibility, and the ability to encapsulate both lipophilic and hydrophilic drugs. In these systems, lipophilic drugs localize within the lipid domains, while hydrophilic drugs are retained in the aqueous core. Niosomes represent more conventional vesicles with controlled-release properties, being typically stabilized with cholesterol to enhance membrane rigidity and reduce permeability [107,108]. Spanlastics differ primarily through the incorporation of an edge activator (e.g., Tween or sodium cholate) instead of cholesterol, imparting greater elasticity, resilience, and deformability to the vesicle membrane. This enhanced flexibility enables them to pass through narrow pores and mucosal channels without losing structural integrity. The term “spanlastic” is derived from a combination of the words “Span”, referring to the non-ionic surfactant used, and “elastic”, referring to the edge activator, which provides elasticity [109,110]. Nanotransferomes are highly deformable, elastic liposomal nanovesicles composed of a bilayer modified with nonionic surfactants (phospholipids and nonionic surfactant), which impart flexibility and enable improved penetration of the active substance through the biological membranes. These vesicles can efficiently transport both hydrophilic and hydrophobic drugs across biological barriers [111,112]. All of these systems have been investigated as promising nanocarriers for buccal drug-delivery applications while being incorporated into pharmaceutical formulations, with the aim of enhancing mucosal permeation, prolonging residence time, and improving bioavailability. Moreover, they can increase the apparent solubility of poorly water-soluble drugs through colloidal solubilization and encapsulation within their lipid-surfactant bilayers, with spanlastics often providing enhanced solubilizing capacity due to their relatively high surfactant content and flexible vesicular structure [104,108,109,112].

The research by Suharyani et al. developed hydrogel mucoadhesive films to be used in recurrent aphthous stomatitis (RAS), incorporating α-mangostin—a natural bioactive compound known for its antifungal, antibacterial, and anti-inflammatory properties, extracted from the *Garcinia mangostana* rind. To improve its solubility and bioavailability, the α-mangostin was complexed with γ-cyclodextrin and incorporated into a hydrogel-based film composed of sodium alginate and chitosan, utilizing the solution casting method. Three distinct formulations were developed for comparative evaluation: films incorporating pure α-mangostin, films containing a physical mixture of α-mangostin and γ-cyclodextrin, and films comprising an inclusion complex of α-mangostin and γ-cyclodextrin. The mucoadhesion profile measurement was performed using a texture analyzer by attaching the film to bovine tongue mucosa. The formulation containing the complex exhibited the greatest mucoadhesive strength (1042.76 ± 0.24 g-force) and longer adhesion duration (49.16 ± 3.28 min) compared to the other. Moreover, an in vivo study in rats indicated that the inclusion complex film demonstrated enhanced anti-RAS activity [67].

To develop buccal films as carriers for fluconazole, sodium caseinates (milk-derived proteins capable of self-assembling into micellar structures) were investigated as film-forming agents. The fluconazole-loaded films were obtained using the spraying method and exhibited hydrogen bonding interactions between the drug molecules and sodium caseinates micelles, which influenced their physicochemical properties. At a sodium caseinates: fluconazole ratio of 1:0.1, the active substance was present in an amorphous state, and the films displayed enhanced flexibility compared to those with higher drug loadings [80].

Another formulation involved mucoadhesive films loaded with fluconazole, sodium caseinate, and mineral clays—magnesium aluminum silicate (MAS) and halloysite (HAL), as polymeric excipients. Investigations highlighted the influence of these materials on the physicochemical and pharmaceutical properties of the resulting films. It was observed that the primary interactions between sodium caseinate, the clays, and fluconazole occurred through hydrogen bonding. MAS demonstrated stronger interactions with fluconazole than HAL, leading to the formation of nanocomposites within the film matrix. The fluconazole-loaded sodium caseinate films exhibited a short dissolution time of approximately 8 min, and the addition of HAL had only a minimal impact on this parameter, but incorporation of MAS markedly extended dissolution to over 1 h. The incorporation of MAS increased the T50% value of the released fluconazole, which maintained its antifungal activity against *C. albicans*. The obtained formulations maintained mucoadhesive properties on porcine mucosa [81].

Mucoadhesive films composed of hydroxypropyl methylcellulose and polyvinyl alcohol, prepared using the solvent casting method, were developed as a novel delivery system for a probiotic extract containing the species *Lactobacillus casei*. The type of polymer used affected the mechanical properties, swelling, and release profile of the designed films. Formulations based on polyvinyl alcohol demonstrated superior tensile strength and greater elongation at break compared to those formulated with hydroxypropyl methylcellulose. However, increasing the hydroxypropyl methylcellulose content led to an increased swelling index and slowed down the extract release. Based on these observations, the formulations comprising 54% hydroxypropyl methylcellulose and 26% polyvinyl alcohol were identified as optimal. Mucoadhesion was assessed with the swelling index of the films which were examined on an agar plate. The films with the proposed composition exhibited inhibitory activity against the growth of *C. albicans*, which—due to the presence of the probiotic extract—makes them a promising candidate for oral candidiasis treatment [68].

Abruzzo et al. developed a buccal delivery system that combines mucoadhesive polymeric matrices prepared via freeze-drying with miconazole-loaded liposomes (LP-MN). The formulation employed hydroxypropyl methylcellulose, hyaluronic acid, and chitosan to create the mucoadhesive matrices. The liposomes (LP) were prepared using L-α-phosphatidylcholine to encapsulate miconazole, aiming to improve drug solubility and enable controlled release (Figure 3). The mucoadhesive properties of the samples were evaluated by measuring their displacement on an inclined agar–mucin surface. Mucoadhesion tests revealed distinct behaviors among the three polymer types. Hyaluronic acid matrices exhibited rapid gelation but limited stability; hydroxypropyl methylcellulose matrices gelled more slowly but maintained greater persistence before dissolving. Although hyaluronic-acid-based formulations absorbed more water than hydroxypropyl methylcellulose matrices, they displayed lower mucoadhesion due to the negatively charged carboxyl groups in hyaluronic acid, which cause electrostatic repulsion with sialic acid residues and sulfate groups in mucin. In contrast, hydrated hydroxypropyl methylcellulose chains could interpenetrate mucin networks and form multiple hydrogen bonds, enhancing adhesion. Despite its lower hydration, the chitosan formulation maintained its shape and remained firmly in place throughout the experiment, with no displacement observed, indicating superior mucoadhesive strength. This is attributed to strong electrostatic interactions between the protonated amino groups of chitosan and the sialic and sulfonic acid groups in the mucosal layer. Matrices composed solely of the polymers (hyaluronic acid and hydroxypropyl methylcellulose) traveled the shortest distances within the same timeframe. Conversely, matrices loaded with LP and LP-MN covered longer distances, suggesting lower mucoadhesive capacity. These results indicate that the reduced hydration capacity of matrices containing LP and the drug resulted in decreased mucoadhesion. This finding suggest that the reduced hydration capacity of the matrices in the presence of both the LP and the drug resulted in diminished mucoadhesive performance. However, despite their higher lipophilicity and lower hydration capacity, chitosan-based matrices with LP and LP-MN maintained similar adhesion to the mucin-agar plate as matrices made only of the polymer, with no displacement observed. These formulations were considered the best candidates for ensuring prolonged residence on the buccal mucosa after application. Antifungal tests against *C. albicans* showed that both chitosan- and hyaluronic-acid-based formulations nearly completely inhibited fungal growth. Notably, hyaluronic-acid-based matrices released a higher amount of miconazole, while chitosan-based matrices released less drug, maintaining similar effectiveness. This highlights the antifungal properties of chitosan [82].

Fluconazole-loaded solid lipid nanoparticles (FLU-SLNs; Figure 4) were incorporated into pectin-based mucoadhesive buccal films. Solid lipid nanoparticles (SLNs) are promising for enhancing drug permeability across the buccal mucosa; however, their use in mucosal delivery is limited by poor retention, as they are easily removed by saliva, swallowing, and oral movements. To address these limitations, the study proposes incorporating SLNs into a mucoadhesive matrix. The FLU-SLNs were prepared from Tween 80 (amount ranging from 2.0 to 2.2 g) and glyceryl monostearate (amount ranging from 1.8 to 2.0 g) utilizing the hot homogenization technique. A decrease in the particles’ size was observed with increasing content of glyceryl monostearate, followed by an increase in particle size once its level exceeded a certain threshold. Four formulations exhibiting small particle sizes (<131.7 nm) and acceptable polydispersity index (<0.5) values were selected for the preparation of the films. The solvent casting method was then used to produce the pectin- based films (with a pectin content 3.8–4.0). FTIR spectra indicated that hydrogen bonds may potentially form between the pectin-based matrix and the components of the FLU-SLN. The mucoadhesive properties of the films were evaluated with a texture analyzer and porcine buccal mucosa, measuring the detachment force from mucin-coated surfaces, which indicated optimal adhesion for prolonged mucosal retention ensuring fluconazole sustained release over 6 h. The formulation composed of pectin, Tween 80, and glyceryl monostearate in ratios of 4:2:2, respectively, showed the highest mean W_ad_ and F_max_ values. This enhancement is consistent with the presence of hydroxyl groups in glyceryl monostearate and carboxyl groups in pectin, both of which facilitate interactions with mucin and support the mucoadhesive performance of the films. All designed films exhibited anti-*Candida* activity, with inhibition zone diameters that did not differ significantly among the formulations [83].

Sodium-alginate-based mucoadhesive films were developed as a platform for the targeted delivery of antifungal plant lectins (a class of carbohydrate-binding proteins) against *C. albicans*. Two lectin compounds were used in the designed research: the one designated as ConBr isolated from *Canavalia brasiliensis* seeds, exhibiting immunomodulatory and antinociceptive activities, and the other designated as MaL derived from *Machaerium acutifolium* seeds, demonstrating antinociceptive, anti-inflammatory, and antifungal effects. However, the therapeutic use of proteins in the oral cavity faces challenges such as enzymatic degradation by salivary proteases and other factors limiting their bioavailability. To address these limitations, a formulation strategy relying on mucoadhesive film preparation was explored to enhance drug stability and their effectiveness in the oral environment. The films were prepared using the solvent casting method. Freshly excised bovine jugal mucosa was used for ex vivo mucoadhesion testing with a texture analyzer. As a result, the films containing ConBr showed improved mucoadhesive properties. ConBr-loaded films exhibited the highest maximum detachment force (0.56 ± 0.04 N), whereas the remaining formulations showed comparable adhesive strength (0.48 and 0.47 N). The antifungal activity of ConBr and MaL lectins was evaluated using a candidiasis model in *Tenebrio molitor* larvae, infected with a clinical oral isolate of *Candida albicans*. Untreated larvae exhibited a median survival of 1.5 days with an overall survival rate of 40%. Both lectins demonstrated prophylactic activity, resulting in survival rates of 80–90%; however, in therapeutic assays, only MaL produced a statistically significant improvement in larval survival (70%) [69].

Mucoadhesive buccal films combining miconazole nitrate and lidocaine hydrochloride were designed to provide both antifungal and analgesic effects for treating oropharyngeal candidiasis. Miconazole was encapsulated into microparticles made of chitosan and various anionic polymers—alginate, κ-carrageenan (κC), λ-carrageenan (λC), or sodium lauryl sulfate (SLS), using a spray-drying method. The obtained microparticles were then embedded into a film matrix composed of hydroxypropyl methylcellulose and gelatine, with sorbitol serving as a plasticizer, containing lidocaine. Chitosan–alginate microparticles demonstrated the highest encapsulation efficiency for miconazole (97.15%), while the lowest (48.88%) for chitosan-SLS formulation. The effectiveness of miconazole encapsulation was higher in the chitosan–alginate carrier, probably due to the high reactivity of the amino and carboxyl groups of the polymers, which may interact with the drug. Dissolution studies revealed a faster release of lidocaine (up to 12 h) and a prolonged release of miconazole (up to 24 h) from the chitosan–alginate particles. Mechanical testing showed that films containing also chitosan–alginate microparticles exhibited the highest tensile strength and the lowest elongation at break, indicating a more rigid structure due to strong polymer interactions. Swelling experiments revealed that films with chitosan-SLS microparticles possessed the highest swelling index, correlating with faster drug release rates. These data showed that films containing chitosan–alginate, chitosan-κC, and chitosan–λC microparticles remained intact for 24 h, whereas systems with chitosan-SLS reached maximum swelling at approximately 6 h, followed by complete disintegration. Mucoadhesive testing showed that all formulations were characterized by a favorable adhesion profile, although microparticle incorporation reduced adhesiveness compared to unloaded films. Antifungal activity was confirmed through halo zone tests against *C. albicans*, where the chitosan–alginate formulation maintained inhibitory effects for up to 24 h (compared to SLS, which lost activity after 8 h), which supports the antifungal role of alginate. Differential scanning calorimetry and X-ray diffraction analyses showed that both miconazole and lidocaine hydrochloride existed in an amorphous form within the films, likely due to the spray-drying process, which may enhance their solubility and bioavailability [84].

Posaconazole-loaded buccal films made from sodium alginate and low-methoxyl pectin were developed by crosslinking utilizing calcium carbonate (CaCO_3_) and glucono-δ-lactone to adjust their mechanical strength and drug release profile. The presence of pectin improved flexibility, mucoadhesion, and antifungal activity, while the crosslinking process enhanced mechanical properties and stability but reduced mucoadhesiveness and antifungal effectiveness. Mucoadhesive strength was assessed using porcine buccal mucosa, and antifungal activity was evaluated against *C. albicans*, *C. krusei*, and *C. parapsilosis*. The obtained results showed that the crosslinking process led to a significant decrease in both mucoadhesiveness and antifungal activity against the tested *Candida* species. However, the modification significantly improved the mechanical strength and stability of the designed formulations, enabling sustained drug release. Among the designed formulations, the one considered to be the most optimal for its mucoadhesive, mechanical, and antifungal properties, with prolonged POS release, consisted of sodium alginate and pectin crosslinked by CaCO_3_ and glucono-δ-lactone in a 0.05:0.19 ratio [85].

Mucoadhesive buccal films containing nystatin, hydrocortisone acetate, and lidocaine hydrochloride were developed to provide a comprehensive treatment for oral candidiasis. The films were prepared utilizing hydroxyethyl cellulose (2.5% or 3%) and xanthan gum (2.8% or 3%) by a solvent casting method with varying concentrations of the polymers. To improve the solubility of the nystatin, either Tween 80 or Cremophor RH40^®^ was added to the formulation. Propylene glycol was added as a plasticizer in a concentration of 6% or 10%. Mucoadhesion was evaluated utilizing a texture analyzer and a bioadhesion test assembly, using porcine buccal mucosa. Xanthan gum-based films (3%) with the addition of Tween 80 and propylene glycol (6%) were assessed as the most optimal due to their highest antifungal activity, amount of released nystatin, and lowest erosion matrix. In vitro release studies demonstrated that the films successfully extended the release of nystatin, while hydrocortisone acetate and lidocaine hydrochloride were released more rapidly, offering immediate anti-inflammatory and analgesic effects. Antifungal activity was tested against *C. albicans* using the disk diffusion method [86].

The bilayer buccal films were designed for the controlled delivery of ciclopirox olamine in the oral cavity. The mucoadhesive layer was made of polyethylene oxide, chosen for its strong adhesion to the buccal mucosa. In contrast, the backing layer used Eudragit^®^ NM 30D, a hydrophobic polymer that directs drug release toward the mucosal surface. The films were produced using the solvent casting method. Their swelling properties and erosion behaviors were assessed by measuring water uptake and matrix erosion or dissolution. The formulation was further tested through in vitro and ex vivo studies, along with pharmacokinetic and efficacy evaluations in a rabbit model infected with *C. albicans*, confirming effective drug delivery and therapeutic potential [87].

Mucoadhesive multilayer buccal films were developed using chitosan and pectin to form polyelectrolyte complexes (PECs) for controlled clotrimazole delivery. The films were created via a solvent casting method with a layer-by-layer deposition technique, incorporating clotrimazole into either the chitosan layer, the pectin layer, or both, resulting in three different formulations: F1–F3. F1 consisted of 50% chitosan and 50% pectin, F2 contained 66.7% chitosan and 33.3% pectin, and F3 was made of chitosan alone. In vitro drug release studies exhibited pH-responsive behavior, with faster drug release at acidic pH (4.8) compared to neutral pH (6.8), corresponding to the pH conditions of the oral cavity during infection. F1 demonstrated pH-responsive release, with about 80% of the drug released after 24 h at pH 6.8. Lowering the pH to 4.8 sped up drug release, reaching the same 80% within just 4 h. A burst release (~40% within 15 min at pH 4.8) was observed, primarily due to the presence of clotrimazole in the PC layer. The physicochemical properties of chitosan, such as increased solubility and quicker disintegration in acidic conditions, likely contributed to its faster release. F2 showed a prolonged, pH-independent release profile, with only 56–62% of clotrimazole released over 24 h, regardless of pH. Over 80% of the drug remained unreleased after 24 h, due to strong ionic interactions within the polymer matrix, making it resistant to pH changes. The minimal surface-associated drug fraction resulted in negligible burst release, which might limit its effectiveness for buccal use. F3 also exhibited pH-responsive release behaviors similar to F1, with approximately 80% of the clotrimazole released at pH 6.8 after 24 h and a faster release under acidic conditions. The burst effect was less prominent than in F1, with about 12% released after 15 min at pH 4.8. It was influenced by the acid-sensitive disintegration of the chitosan layer, but to a lesser extent than in F1. Mucoadhesive properties were tested using a texture analyzer with porcine buccal mucosa. Swelling and erosion studies confirmed that the pectin layer aided initial drug release, while the chitosan layer supported sustained release. Mucoadhesive strength tests showed that F1 and F2 had a greater mucoadhesion profile than F3 and placebo films, thanks to optimal swelling and polymer–mucin interactions. Antifungal activity against *C. albicans*, *C. krusei*, and *C. parapsilosis* revealed that all clotrimazole-loaded films produced significant inhibition zones, with greater activity in chitosan-based formulations, indicating a synergistic effect between chitosan and clotrimazole. Cytotoxicity tests with human gingival fibroblasts indicated that the films were biocompatible at concentrations up to 0.2%, with higher concentrations reducing cell viability, likely due to the presence of free polyelectrolytes [88].

Mucoadhesive buccal films containing clotrimazole and graphene oxide were prepared using polyelectrolyte complexes of chitosan and alginate. Graphene oxide is an amphiphilic, high-surface-area material with significant potential as a functional carrier in drug delivery systems. In this study, it was incorporated to evaluate its influence on drug release kinetics and antifungal activity against *C. albicans*. Physicochemical characterization showed that the addition of graphene oxide altered the moisture uptake and swelling behavior of the films, resulting in a faster release of clotrimazole. Films containing graphene oxide exhibited enhanced drug release compared with graphene-oxide-free films, likely due to its ability to disrupt hydrophobic and electrostatic interactions between clotrimazole and the polymer matrix. An increase in antifungal activity against *C. albicans* was observed upon incorporation of 0.04% graphene oxide. Notably, raising the graphene oxide content to 0.09% did not further enhance antifungal efficacy, as the films showed similar activity to those containing 0.04%, suggesting that electrostatic and hydrophobic interactions between graphene oxide and clotrimazole may also modulate the drug’s antifungal performance. In vitro antifungal assays confirmed that films containing graphene oxide produced larger inhibition zones than those without the compound [89].

Miconazole-loaded buccal films created through 3D printing were designed to provide a child-friendly antifungal treatment. In the production process, a semi-solid extrusion method was used to produce films from a mixture of zein and polyvinylpyrrolidone, which served as the drug carrier. Mucoadhesion tests were conducted with a texture analyzer, using porcine mucosa as the test surface. Results showed that higher polyvinylpyrrolidone concentrations enhanced the adhesive properties of the films. The mechanical strength was sufficient for the intended application, with the 40:60 zein–zein-polyvinylpyrrolidone formulation displaying the greatest deformation capacity. The antifungal activity of the designed films was tested against *C. albicans*, demonstrating significant inhibitory effects. The formulation provided a sustained release of miconazole, with approximately 80% released within 2 h [90].

Mucoadhesive buccal films containing atorvastatin-loaded propylene-glycol-integrated liposomes (ATV/PG-Lip) were developed utilizing 3D printing technology to target fluconazole-resistant *C. albicans* infections. Although primarily used as a cholesterol-lowering agent, atorvastatin has demonstrated antifungal potential (repurposing of the active substance) based on inhibiting the production of ergosterol in the cell wall of fungi. A liposome formation strategy was used to improve the solubility and enhance the permeability of atorvastatin. The films were composed of a polymer blend of chitosan, polyvinyl alcohol, and hydroxypropyl methylcellulose. Mucoadhesive strength was assessed utilizing a texture analyzer, using chicken pouch membranes as the mucoadhesive layer. The in vitro antifungal activity was confirmed by measuring the minimum inhibitory concentration (MIC), with ATV/PG-Lip showing a higher MIC than free atorvastatin, likely due to encapsulation of the active substance within liposomes. Scanning electron microscopy revealed significant morphological damage to fungal cells treated with ATV/PG-Lip. In vivo studies using a rabbit model of oral candidiasis demonstrated that the designed films significantly reduced fungal infection and inflammation, as indicated by lower levels of pro-inflammatory cytokines TNF-α and IL-6 [70].

Bilayered buccal tablets containing natamycin were developed using a 3D printing method, consisting of a mucoadhesive layer with Carbopol^®^ 974P NF to ensure adhesion to the buccal mucosa, and a drug-loaded layer with varying concentrations (10–40% *w*/*w*) of hydroxypropyl methylcellulose to control the release rate of natamycin. Additionally, another batch incorporating erythrosine (a synthetic red food coloring) as a control marker was formulated to track the in vivo distribution of the released contents in the oral cavity (referring to tablets containing the active substance). In vitro studies showed that higher hydroxypropyl methylcellulose concentrations reduced the rate of natamycin release, and the work of adhesion decreased with increasing hydroxypropyl methylcellulose levels (measured using a texture analyzer). A volunteer study evaluated the distribution of the drug within the oral cavity, revealing that the formulation maintained natamycin concentrations above MIC for *C. albicans* in most regions, except the sublingual area, which showed lower concentrations due to increased salivary flow [91].

To enhance the localized delivery of nystatin, mucoadhesive buccal tablets containing purified cashew gum as both a binder and mucoadhesive agent were developed. Two batches of tablets, namely MT1 and MT2, were produced via direct compression. Each tablet contained 500,000 IU of nystatin, purified cashew gum, flavoring agents, and, in the case of MT2, a lubricant (magnesium stearate). Mucoadhesion tests using porcine buccal mucosa showed that MT1 and MT2 adhered for up to 3 and 4 h, respectively, using a two-pan balance system. Batch MT1 experienced rapid initial swelling that slowed over time, while MT2 exhibited slower swelling, likely because the magnesium stearate hindered water absorption and polymer relaxation. The high porosity and low surface tension of cashew gum promoted water uptake, swelling, and mucoadhesive interactions with mucin. In MT1, greater initial swelling trapped more water within the polymer matrix, reducing drug release and slowing erosion, whereas the lower swelling of MT2 led to faster early-stage erosion. Over time, swelling in MT1 decreased, and erosion increased. In vitro release studies confirmed that both formulations released enough nystatin to inhibit the growth of *C. albicans* [92].

A series of mucoadhesive buccal tablets containing chlorhexidine digluconate was formulated using hydroxypropyl methylcellulose, combined with poloxamer 407 and selected polyols, such as sorbitol, mannitol, and xylitol, to modulate swelling behavior and drug release profiles. Each group was prepared with two different ratios of polymers (poloxamer 407 to hydroxypropyl methylcellulose) and two different ratios of polyols. Mucoadhesive properties were evaluated based on the residence time of the tablets on the mucosal tissue under simulated physiological conditions. For this purpose, chicken crop tissue served as a biological model. The results showed that increasing concentrations of poloxamer 407 and polyols decreased the swelling index and accelerated the release of chlorhexidine digluconate. The most promising formulations were those designed in the poloxamer 407/hydroxypropyl methylcellulose/polyol ratio 3:1:2. The antimicrobial efficacy of chlorhexidine digluconate was confirmed against *C. albicans* biofilms, and biocompatibility studies using HEK293 human cell lines indicated that the formulations were non-toxic at therapeutic concentrations [93].

Recent advances include the development of nystatin-loaded mucoadhesive spanlastic hard candy lozenges, an innovative dosage form for managing oral candidiasis, particularly advantageous in the pediatric population, as the lozenges offer a convenient and palatable drug delivery system. Spanlastics were designed to improve drug permeation, stability, and release profiles across biological membranes (Figure 5). Such a strategy is especially beneficial for nystatin, a Biopharmaceutics Classification System (BCS) Class IV drug characterized by poor aqueous solubility and low permeability, which significantly hinders its clinical use. The spanlastics were prepared using Span 60, Span 80, Tween 20, and sodium deoxycholate (Figure 5), before being incorporated into hard candy lozenges formulated with β-cyclodextrin, mannitol, acacia gum, xanthan gum, corn syrup, and microcrystalline cellulose. The most effective spanlastic formulation was identified as the one combining Span 80 with sodium deoxycholate. Correspondingly, the optimal lozenges were produced from the batch with the concentrations of corn syrup (400 mg) and xanthan gum (250 mg). The mucoadhesion strength of the lozenges was measured using a modified balance method. The swelling index of the lozenges increased with higher concentrations of corn syrup compared to xanthan gum. This was probably due to the hygroscopic nature of corn syrup, which enhances its ability to attract and hold water within the lozenge matrix. The formulations demonstrated superior in vivo antifungal efficacy, shown by a significant reduction in colony-forming units compared to the marketed nystatin oral suspension. Additionally, the rats treated with the optimized lozenges displayed normal oral tissue morphology at the end of the study, unlike those receiving the commercial one. The obtained results emphasize the potential of nanotechnology-based mucoadhesive lozenges as a better alternative to marketed formulations, offering improved antifungal efficacy, better acceptability, and longer mucosal residence [94].

Mucoadhesive nanoparticles containing miconazole were developed using thiolated xanthan gum to improve drug permeation, produced via high-pressure homogenization. Five formulations were prepared with varying concentrations of trisodium polyphosphate to obtain nanoparticles. Among these, the formulation with a 1:1 ratio of thiolated xanthan gum to trisodium polyphosphate was identified as optimal, as it exhibited the smallest particle size (87 ± 2 nm). A key innovation was the thiolation of xanthan gum using mercaptoacetic acid to enhance mucoadhesive properties. In vitro release studies of miconazole in phosphate buffer revealed that thiolated xanthan gum nanoparticles provided a more consistent release profile compared to conventional miconazole dispersion. To evaluate mucoadhesive properties, the ex vivo adhesion time of thiolated xanthan gum with nanoparticles was measured using sheep intestinal mucosa as a biological membrane. Results indicated that the formulation had a significantly longer mucoadhesion profile compared to unmodified xanthan gum, an effect attributed to free thiol groups forming disulfide bonds with cysteine-rich domains of mucin. Antifungal activity against *C. albicans* assessed via agar diffusion demonstrated that thiolated xanthan-gum-based nanoparticles were considerably more effective than the conventional miconazole gel, achieving complete inhibition at a lower drug concentration than that contained in the commercially available counterpart. The animal model, albino rats, was employed to evaluate the pharmacokinetics of miconazole, revealing a 4.5-fold enhancement in the drug’s bioavailability [95].

Hydrogel patches composed of thiolated κ-carrageenan and pectin were developed as a new mucoadhesive delivery system. To create the thiolated derivative, κ-carrageenan was chemically modified by grafting acrylic acid, followed by functionalization with L-cysteine and 3-mercaptopropionic acid. Triamcinolone acetonide, a corticosteroid with strong anti-inflammatory properties, was incorporated into the patches either directly or after encapsulation in poly(lactic-co-glycolic acid) nanoparticles. The drug release profiles indicated that patches containing the free drug released the active substance faster, while patches with nanoparticles released the drug in a slower manner over the same period of time. Ex vivo mucoadhesion tests on porcine buccal mucosa demonstrated enhanced mucoadhesive properties resulting from the thiolation of κ-carrageenan, which facilitates disulfide bond formation with mucosal glycoproteins. Swelling studies showed that the patches could absorb significant amounts of fluid, benefiting both drug release and mucosal adhesion. Antifungal activity tests revealed that the drug-loaded patches inhibited *A. fumigatus* and *A. flavus* growth, while the drug-free patches did not show microbial inhibition zones [72].

### 5.2. Semi-Solid Drug Dosage Forms

Another major category of mucoadhesive formulations includes semisolid dosage forms, especially gels, which can be tailored in terms of rheological and adhesive properties to improve therapeutic outcomes. Thermosensitive gels have gained significant interest as buccal drug delivery systems for treating oral lesions. They are typically applied as liquids at room temperature and then undergo a sol–gel transition upon contact with body temperature, forming an in situ gel right at the application site. Applying such an approach provides prolonged residence on the mucosal surface, shielding the lesion from salivary clearance and enabling sustained, localized drug release, while also acting as an easy-to-administer drug dosage form [106].

The freeze-dried polyelectrolyte complex-based hydrogels containing atorvastatin calcium have been developed to improve drug release profiles and facilitate effective buccal delivery. The hydrogels were formulated using low-molecular-weight chitosan as the cationic component, combined with various anionic polysaccharides such as tragacanth gum, xanthan gum, low-methoxy amidated pectin, and κ-carrageenan. The lyophilized hydrogels demonstrated strong mucoadhesive properties, ensuring extended retention at the application site. Characterization of the formulations showed that freeze-drying increased the amorphization of atorvastatin, leading to enhanced drug release. The lyophilized hydrogels released significantly more atorvastatin compared to their non-lyophilized versions. The mucoadhesive strength of the hydrogels was assessed using a texture analyzer. Additionally, commercial Elugel^®^ was used as a positive control, with cellulose paper serving as a negative control. The results indicated that the freeze-dried hydrogels had greater mucoadhesive strength than their non-lyophilized counterparts, suggesting better potential for prolonged retention and effectiveness in treating oral candidiasis. The antimicrobial activity of the formulations was tested against various *Candida* species, including *C. albicans*, *C. krusei*, and *C. parapsilosis*. Notably, they showed strong antifungal activity against *C. albicans*, exceeding that of a commercially available clotrimazole cream. However, activity against *C. krusei* and *C. parapsilosis* was less impressive, with some formulations demonstrating limited or no efficacy. Importantly, the formulations did not harm beneficial oral microbiota such as *Lactobacillus brevis*, indicating a favorable selectivity profile [71].

Novel topical caspofungin (2%) formulations, including mouthwashes, hydrogels, and pastes, were developed using mucoadhesive polymers such as hyaluronic acid, chitosan, and thermosensitive poloxamer 407. Mucoadhesion ex vivo was measured by assessing the resistance or tension utilizing a simple experimental setup. It was concluded that gels or pastes are preferable for localized lesions, while mouthwashes are more appropriate for extensive lesions or large mucosal areas. The chitosan formulation showed by far the greatest retention in both buccal and sublingual mucosa, as well as the strongest mucoadhesive force, along with the best extensibility for easy oral application. The Poloxamer^®^ P407-based hydrogel showed the highest drug release (about 70% within 28 h), due to its micellar properties, which improve drug solubility. Additionally, considering the thermosensivity of the polymer, it can be administered as a spray, especially for extensive lesions or hard-to-reach areas. The antifungal activity of the formulations was tested using a modified disk diffusion method against *Candida* strains (*C. albicans*, *C. auris*, *C. glabrata*, *C. tropicalis*, *C. parapsilosis*). All formulations were active against the tested species, except for *C. auris.* The chitosan-based hydrogel was the most effective, as the antifungal properties of chitosan synergistically enhanced caspofungin activity, resulting in larger inhibition zones [96].

A thermosensitive, mucoadhesive gel was developed as a dual-drug delivery system with clindamycin and fluconazole, to improve localized treatment of periodontal infections, as bacterial and yeast pathogens often coexist and act synergistically. Therefore, clindamycin-loaded niosomes along with fluconazole-loaded solid lipid nanoparticles (SLNs) were prepared. The structure of the niosims allows them to encapsulate both hydrophilic and hydrophobic drugs (Figure 6). SLNs, on the other hand, consist of solid lipids dispersed in water and stabilized with a surfactant. The choice of niosomes for clindamycin and SLNs for fluconazole was based on the distinct physicochemical properties of each drug. Clindamycin, being more hydrophilic, is more effectively encapsulated in the aqueous core of niosomes, while the lipophilic fluconazole is better suited for incorporation into the lipid matrix of SLNs. Niosomal formulations of clindamycin demonstrated higher drug loading efficiency and a more sustained release, whereas SLNs containing fluconazole had smaller particle sizes and a relatively faster release. Both formulations were dispersed within a Pluronic^®^ F127 hydrogel, which remains a solution at room temperature but transforms into a mucoadhesive gel when injected into periodontal pockets. The antimicrobial activity was tested against *S. aureus*, *L. casei*, and *C. albicans*, and the formulation proved effective against all three pathogens [97].

A thermosensitive mucoadhesive hydrogel was developed for the localized delivery of a novel anti-*Candida* peptide, engineered based on a natural molecule originally found in the hemolymph of the freshwater crayfish *Procambarus clarkii*, providing a promising alternative to combat the rising resistance to conventional antifungal drugs. The hydrogel matrix was made from a graft copolymer of xanthan gum and poly(N-isopropylacrylamide), providing temperature-sensitive viscoelasticity and pseudoplastic behavior. The formulation exhibited physicochemical stability and optimal mucoadhesive properties, and, additionally, compatibility with cells and a favorable release profile of the peptide, with approximately 90% released within 24 h. Importantly, the hydrogel significantly inhibited *C. albicans* growth compared to the control [98].

Mucoadhesive nanogels containing fluconazole were designed to improve the solubility and targeted delivery of the active substance. Nanogels are defined as hydrogels with a 3D, tunable porous structure and particle sizes ranging from 20 to 250 nm, being sensitive to pH and temperature, which provides them with controlled drug release properties. The formulation was created by first forming a fluconazole– sulfhydryl-β-cyclodextrin (SH-β-CD) inclusion complex, which was then incorporated into nanogels formed through the interaction of opposite charge polymers, carbopol 940 and gelatin in different molar masses. Cyclodextrins were employed to enhance the solubility of the fluconazole and combined with free sulfhydryl groups to improve mucoadhesive properties (thiolation process). The nanogels formulated with a higher amount of CA-940 and subsequently loaded with the inclusion complexes exhibited the most promising characteristics. Mucoadhesive properties were tested using goat buccal mucosa, showing a greater increase in mucoadhesion for the SH-β-CD-based nanogels compared to the non-complexed ones. In vitro permeation studies exhibited significantly enhanced permeation and first-order concentration-dependent drug release, while cytotoxicity studies on Caco-2 cell lines confirmed the safety of the designer nanogels. Nanogels loaded with FL-SH-β-CD complexes showed 18-fold improvement in mucoadhesion on the goat buccal mucosa compared to simple nanogels [99].

Buccal delivery systems in the form of gel or films containing miconazole-loaded solid lipid microparticles (SLMs) were developed to improve drug retention and therapeutic effectiveness. SLMs exhibited optimal physicochemical stability and high loading capacity for lipophilic or poorly water-soluble active substances, thus enhancing their bioavailability. The SLMs were prepared using cetyl decanoate and 1-hexadecanol, resulting in spherical particles highly loaded with miconazole in the amorphous state, with a melting point compatible with the physiological temperature of the oral cavity. The microparticles were incorporated into buccal films or gels as a final drug dosage form to facilitate easier dosing and application. The gel formulation was composed of hydroxyethylcellulose, polyvinylpyrrolidone K90, and trehalose. The films were prepared using the solvent casting method from aqueous gels also containing polyvinylpyrrolidone K90 and hydroxyethyl cellulose, with either trehalose, acting as a potential antifungal agent, or limonene as a penetration enhancer. It was observed that formulations with trehalose or trehalose and limonene exhibited the highest folding endurance. Mucoadhesive properties were assessed using a modified balance method. Despite their lipid content, the films retained the ability to absorb surrounding fluids and swell, forming a dense gel layer that effectively stabilizes the SLMs at the target site. Ex vivo studies utilizing porcine buccal tissue demonstrated that SLM formulations increased miconazole accumulation threefold compared to the commercial 2% miconazole gel (Daktarin^®^) in the porcine buccal mucosa [100].

A mucoadhesive oral gel was formulated by incorporating fluconazole-loaded nanotransfersomes (FL-NTFs; Figure 7) to improve drug delivery and retention in the oral cavity. The FL-NTF were prepared using the thin-layer evaporation method of lecithin, sesame oil, and Tween 20. The formulation was optimized to assess how concentrations of the utilized compounds affected vesicle size, entrapment efficiency (EE%), antifungal activity (zone of inhibition), and oral ulcer index in a rat model. The FL-NTFs were integrated into a cross-linked hyaluronic acid hydrogel. Rheological tests revealed that the gel exhibited thixotropic and pseudoplastic behaviors, which are advantageous for oral application. Ex vivo permeation studies using sheep buccal mucosa exhibited improved permeation of fluconazole from the gel containing FL-NTFs compared to fluconazole suspension and fluconazole in powder form incorporated into the hydrogel used as controls. The antifungal activity was evaluated with disk diffusion assays against *C. albicans*, where the gel formulation showed the largest inhibition zone. In vivo experiments with an immunocompromised rat model of oral candidiasis demonstrated that the FL-NTFs-loaded hydrogel significantly reduced the ulcer index compared to other formulations, indicating enhanced therapeutic efficacy [101].

### 5.3. Liquid Drug Dosage Forms

Liquid dosage forms remain among the less frequently employed mucoadhesive drug delivery systems, due to limited adhesion, faster saliva wash out, and challenges in maintaining accurate dosing. However, ongoing research efforts are also focused on developing such formulations [102,103]. A microemulsion containing miconazole was developed to enhance the bioavailability, solubility, and retention of miconazole for buccal administration, utilizing oleic acid as the lipophilic phase, and Tween 20 and polyvinylpyrrolidone 400 (PEG 400) to improve drug solubilization and, thus, bioavailability. The optimized microemulsion consisted of 2% miconazole with oleic acid (5%), Tween 20 (40%), PEG 400 (20%), and water in specific ratios. Rheological analysis confirmed pseudoplastic behavior, which is favorable for buccal application, while in vitro studies demonstrated the sustained release profiles of miconazole [102].

A mucoadhesive oral film-forming spray was formulated for localized delivery of fluconazole. The formulation combined the polymers: hyaluronic acid, polyvinyl alcohol, xanthan gum, and polyethylene glycol 400, in different ratios. The mucoadhesive properties of the designed formulations were assessed using a turbidimetric method, measuring the interaction between the film-forming solutions and mucin by monitoring changes in turbidity, which reflect mucoadhesion strength (an increase in turbidity indicates stronger adhesion), thus providing a quantitative measure of how the spray formulations adhered to the mucosal surface. The results showed that the hyaluronic acid solution had better mucoadhesive properties than the one prepared utilizing xanthan gum, moreover, each of them performed improved properties than the polyvinyl alcohol. Additionally, increasing the concentration of PEG 400 (10–30%) reduced mucoadhesion. Combining hyaluronic acid with xanthan gum enhanced mucoadhesion compared to hyaluronic acid alone. Therefore, a formulation consisting of hyaluronic acid (0.3% *w*/*v*), polyvinyl alcohol (0.3% *w*/*w*), xanthan gum (0.1% *w*/*v*), and PEG 400 (10%) was chosen as the optimal formulation, which additionally exhibited a larger inhibition zone against *C. albicans* compared to the control group, possibly due to the barrier properties of the used polymers [103].

## 6. Conclusions and Future Perspectives

In recent years, significant progress has been made in developing mucoadhesive antifungal formulations for the treatment of oral candidiasis—a pressing clinical problem, particularly among immunocompromised patients. Providing mucoadhesion, and thus improved local drug delivery, represents a crucial aspect of therapy. Considering the potential side effects and interactions associated with systemic antifungal treatment, delivering the drug directly to the infection site is a more favorable approach for patient care.

Solid drug dosage forms constitute the major class of mucoadhesive preparations; notable among them are mucoadhesive polymer-based films and tablets, although oral gels are also commonly formulated. Among solid, semi-solid, and liquid dosage forms, key factors influencing clinical potential include the physicochemical properties of the polymer matrix, the degree of hydration and swelling, the strength and type of mucoadhesive interactions, and the ability to achieve sustained, localized drug release. Natural polymers such as chitosan, pectin, alginate, and xanthan gum consistently provide strong mucoadhesion and biocompatibility, while thiolated derivatives and polyelectrolyte complexes further enhance adhesion through electrostatic interactions, hydrogen bonding, and improved polymer–mucin interpenetration.

It should be emphasized that incorporating micro- or nanoparticle-based delivery systems (such as nanogels, microparticles, liposomes, nanotransferomes, niosomes, or spanlastics) into drug dosage forms represents a sophisticated approach. It supports more precise and controlled drug release profiles, prolonged retention, and often improves antifungal efficacy due to the polymer-specific features of these systems. Additionally, the use of micro- and nanocarriers provides a promising approach to overcoming the limited water solubility of many antifungal agents, enhancing their therapeutic effectiveness. Importantly, such carriers may allow the encapsulation of both hydrophilic and hydrophobic drugs, greatly expanding the spectrum of therapeutics that can be delivered through mucoadhesive systems and highlighting their relevance for addressing diverse clinical needs. Furthermore, designing multifunctional mucoadhesive systems that not only enable drug delivery but also provide additional protective barriers for both the drug and the oral mucosa has emerged as a growing trend.

Importantly, the substantial methodological variability among studies—encompassing differences in testing substrates, mucoadhesion models, release media, and in vivo systems—significantly limits the ability to make direct quantitative comparisons. This heterogeneity reflects the absence of standardized, universally accepted protocols for evaluating topical mucoadhesive formulations, as each method carries its own advantages and limitations. Consequently, there is a clear need to establish harmonized evaluation guidelines at the early stages of formulation development to ensure reproducibility, improve comparability, and accelerate the translation of advanced oral antifungal delivery systems into clinical practice. The future directions suggest that progress in managing oral candidiasis will depend on mucoadhesive, multifunctional, and patient-specific delivery systems capable of withstanding the dynamic oral environment while delivering localized, potent, and sustained antifungal action. Continued interdisciplinary collaboration combining polymer chemistry, nanotechnology, microbiology, and clinical science will be vital in translating these innovations into practical applications.

## Figures and Tables

**Table 1:** Types of oral fungal infections [7,10,17,18,19,20,21,22,23,24,25].

Type	Causative Agent(s)	Common Symptoms	Predispositions and Risk Factors
**Pseudomembranous candidiasis** **(“oral thrush”)**	*C. albicans*, *C. glabrata*, *C. krusei*,	White, creamy, curd-like plaques on the buccal mucosa, tongue, or palate, which can be wiped off, leaving a red or bleeding base.	Immunosuppression, diabetes, dentures, broad-spectrum antibiotics, age: neonates and the elderly.
**Erythematous (atrophic) candidiasis**	*C. albicans*, *C. glabrata*,	Red, painful areas, especially under dentures or on the tongue. Presents with red, flat lesions, often on the dorsal tongue or palate.	Antibiotic/corticosteroid use, HIV, diabetes, dentures.
**Chronic hyperplastic candidiasis (candidal leukoplakia)**	*C. albicans*	White plaque patches that cannot be wiped off.	Smoking, immunosuppression; requires biopsy due to risk of dysplasia or malignancy.
**Angular cheilitis**	*C. albicans*, *C. glabrata*, *C. dubliniensis*, *S. aureus*	Erythema, maceration, and fissuring at the corners of the mouth. Frequently associated with co-infection with *S. aureus*.	Lip licking, dentures, nutritional deficiency, drooling.
**Median rhomboid glossitis**	*C. albicans*	Erythematous, depapillated area on the midline of the dorsal tongue.	Smoking, corticosteroids, immunocompromise.
**Denture stomatitis**	*C. albicans*, *C. glabrata*, *C. parapsilosis*,	Redness and discomfort of the palatal mucosa beneath dentures are often asymptomatic.	Poor denture hygiene and its continuous wear.

**Table 3:** Examples of mucoadhesive polymers utilized in the pharmaceutical technology [44,45,47,48,49,50,51,52,53,54,55,56,57].

Polymer	Characteristics
***Polymer classification***	
**Natural polymers—polysaccharides**	
**Xanthan gum**	A high-molecular-weight natural polymer produced by the fermentation of carbohydrates by *Xanthomonas campestris*. Consists of a β-(1→4)--D-glucose backbone, with trisaccharide side chains attached to alternating glucose units. Each side chain typically contains a mannose–glucuronic acid–mannose sequence, in which the terminal mannose may be partially acetylated and the internal mannose frequently pyruvylated. The structural features impart a strong anionic character to the polymer. It exhibits very high viscosity even at low concentrations and is readily soluble in water, forming stable, pseudoplastic solutions. High molecular weight, anionic charge, and abundance of hydroxyl and carboxyl groups enable multiple hydrogen bonds and electrostatic interactions with mucin glycoproteins, contributing to effective mucoadhesive behavior.
**Guar gum**	A natural, high molecular weight, hydrophilic, non-ionic, natural polymer, derived from the seeds of the guar plant (*Cyamopsis tetragonolobus*). Composed predominantly of galactomannan—a linear backbone of β-(1→4)-linked mannose units with branched α-(1→6)-linked galactose residues in a ratio of approximately 2:1. The structure imparts high water solubility and a strong ability to swell upon hydration, forming highly viscous colloidal dispersions even at low concentrations. Improves viscosity, swelling capacity, and hydration layer formation in mucoadhesive formulations, thereby strengthening mechanical integrity and contributing positively to mucoadhesive performance, particularly when used in combination with other polymers.
**Chitosan**	A natural, linear cationic polymer composed of β-(1→4)- linked D-glucosamine and N-acetyl-D-glucosamine units; obtained through the partial deacetylation of chitin. The degree of deacetylation determines charge density, solubility, and functional properties. In acidic environment, the primary amino groups (-NH_2_) on the D-glucosamine residues become protonated, giving chitosan a positive charge. In neutral and alkaline environments (pH 7.4 and above), it becomes only poorly soluble or completely insoluble in water. The cationic nature underlies its mucoadhesive behavior, antimicrobial activity, and broad applicability in drug delivery systems. Its positive charge enables it to form strong ionic bonds with the negatively charged mucosal surface. Biodegradable, biocompatible, and non-toxic with anti-inflammatory, antimicrobial, virucidal, and fungicidal properties, as well as the ability to promote tissue regeneration.
**Sodium alginate**	A natural anionic polysaccharide derived from brown seaweed. It consists of alternating blocks of α-L-guluronic acid and β-D-mannuronic acid residues, which enable it to form viscous gels. The mucoadhesive properties result from hydrogen bonds and ionic interactions between its carboxyl groups and mucin in the mucus layer. Upon interaction with multivalent cations, especially calcium ions (Ca^2+^), the polymer forms polyelectrolyte complexes with cationic polymers such as chitosan. Interacts with mucin through hydrogen bonding and electrostatic interactions, contributing to prolonged residence time on mucosal surfaces. Under acidic conditions, protonation of carboxyl groups reduces electrostatic repulsion and leads to polymer precipitation or gel formation, which can slow hydration and drug diffusion. At neutral and alkaline pH, alginate is fully ionized, exhibiting increased solubility, enhanced swelling behavior, and greater chain flexibility.
**Hyaluronic acid**	A naturally occurring, linear polysaccharide composed of repeating disaccharide units of D-glucuronic acid and -acetyl-D-glucosamine. Highly hydrophilic and exhibits excellent biocompatibility, biodegradability, and viscoelasticity. Particularly valued for its potential to bind to mucosal surfaces, primarily through hydrogen bonding and electrostatic interactions with mucin glycoproteins. High molecular weight and polyanionic nature enhance the capacity to form strong physical entanglements with the mucus layer. Upon hydration, swells and forms a viscoelastic gel, which increases residence time at the application site and promotes sustained drug release. Furthermore, hyaluronic acid can also interact with cellular receptors, such as CD44, thereby enhancing the potential for targeted delivery and mucosal tissue regeneration. Characterized by anti-inflammatory and tissue regenerative properties, and, due to its ability to bind water, it moisturizes mucous membranes.
**Hydroxypropyl methylcellulose (HPMC)**	A semi-synthetic, non-ionic cellulose ether derived from natural cellulose obtained by the partial substitution of hydroxyl groups in the cellulose backbone with *methoxy* (-OCH_3_) and *hydroxypropoxy* (–OCH_2_CH(OH)CH_3_) groups. Retains the β-(1→4)-linked D-glucose repeating units characteristic of cellulose, while the degree of substitution and molar substitution determine its solubility, viscosity, gelation temperature, and hydration behavior. Highly hydrophilic and readily hydrates in aqueous environments to form viscous, transparent gels. Non-ionic nature minimizes pH-dependent solubility changes, ensuring consistent performance across physiological pH ranges. The polymer adheres to mucosal tissues primarily through hydrogen bonding and physical entanglement with mucin chains. Upon hydration, swells and forms a gel-like matrix, increasing the surface area for interaction with the mucus layer and providing prolonged residence time and sustained drug release.
**Synthetic polymers**	
**Polivinyl alcohol (PVA)**	A water-soluble polymer obtained by the hydrolysis of polyvinyl acetate, during which acetate groups are replaced by hydroxyl functionalities. Chemical structure consists of a linear carbon backbone of repeating vinyl alcohol units –[CH_2_–CH(OH)]–, with the degree of hydrolysis determining the proportion of hydroxyl to residual acetate groups. The abundance of hydroxyl groups imparts strong hydrophilicity, enabling extensive hydrogen bonding, high film-forming capacity, and excellent mechanical strength. Upon hydration, forms flexible, cohesive gels capable of adhering to mucosal surfaces primarily through hydrogen bonding and physical entanglement, contributing to prolonged residence time and controlled drug release.
**Crosslinked polyacrylic acid** **(Carbomer)**	A high molecular weight, synthetic polymer of acrylic acid with chemical structure consists of repeating acrylate units formed by polymerization of acrylic acid monomers (–CH_2_–CH(COOH)–), with the carboxyl groups providing strong hydrophilicity and ionization under physiological conditions. The chains are crosslinked using allyl ethers of pentaerythritol, sucrose, or propylene, creating a three-dimensional network that swells extensively upon hydration. Mucoadhesive properties arise primarily from the carboxylic acid groups, which create strong hydrogen bonds and electrostatic interactions with the glycoproteins in mucus.
**Methacrylate copolymer**	The group of synthetic polymers composed of various combinations of methacrylic acid and its esters. By adjusting the ratio of monomers, such as methyl methacrylate, ethyl acrylate, and methacrylic acid, the copolymers may be tailored to exhibit specific solubility profiles, pH responsiveness, and functional properties. In mucoadhesive drug delivery, specific grades of methacrylate copolymers, particularly those containing carboxylic acid groups (e.g., Eudragit^®^ RS, E PO), can interact with mucosal surfaces through hydrogen bonding and electrostatic interactions.
**Poloxamers** **(Pluronics**^®^)	A tri-block copolymers composed of poly(ethylene oxide)/poly(propylene oxide)/poly (ethylene oxide) (PEO-PPO-PEO), whose aqueous solutions transition from sol-gel to gel as the temperature rises above the lower critical gelation temperature. There are many different types of Pluronics^®^ available on the pharmaceutical market, differing in the molecular weight of their building blocks and the ratio of hydrophobic to hydrophilic units. Pluronic^®^ 407 (F127) has been widely studied as a drug carrier due to its low toxicity, reverse thermal gelling, high drug carrying capacity, and ability to gel under physiological conditions.
**Others**	
**Gelatin**	A natural, biodegradable, and biocompatible compound derived from the partial hydrolysis of collagen, primarily sourced from animal connective tissues. Consists mainly of polypeptide chains rich in glycine, proline, and hydroxyproline, which confer structural integrity and gel-forming properties. Interacts with mucin glycoproteins through electrostatic interactions, hydrogen bonding, and van der Waals forces. The swelling behavior in aqueous environments contributes to improved adhesion by increasing surface contact with the mucus.

**Table 4:** Review of antifungal mucoadhesive formulations reported in the scholarly research literature by the type of drug dosage form.

*Active* *Substance*	*Drug Dosage Form*	*Polymers*	*References*
***Solid drug dosage forms***			
α-mangostin	Film	Sodium alginate, chitozan	[67]
Fluconazole	Film	Sodium caseinate	[80]
Fluconazole	Film	Sodium caseinte, clay minerals	[81]
Probiotic extract (*Lactobacillus casei*)	Film	HPMC, PVA	[68]
Miconazole	Polymeric matrix with liposomes	HPMC, hyaluronic acid, chitosan	[82]
Fluconazole	Buccal film with solid lipid nanoparticles	Pectin	[83]
Antifungal lectins	Film	Sodium alginate	[69]
Miconazole, lidocaine hydrochloride	Film with microparticles	HPMC, gelatin	[84]
Posaconazole	Film	Sodium alginate, pectin	[85]
Nystatin, hydrocortisone acetate, lidocaine hydrochloride	Film	Xanthan gum, hydroxyethyl cellulose	[86]
Ciclopirox olamine	Bilayer film	Polyethylene oxide, Eudragit^®^ NM 30D	[87]
Clotrimazole	Film	Chitosan, pectin	[88]
Clotrimazole	Film	Chitosan, sodium alginate	[89]
Miconazole	3D printed film	Zein, polyvinylpyrrolidone	[90]
Atorvastatin	3D printed film	Chitosan, PVA, HPMC	[70]
Natamycin	Bilayered buccal tablet	Carbopol^®^ 974P NF, HPMC	[91]
Nystatin	Buccal tablet	Cashew gum	[92]
Chlorhexidine	Buccal tablet	HPMC, poloxamer 407	[93]
Nystatin	Spanlastic hard candy lozenge	Acacia gum, xanthan gum	[94]
Miconazole	Nanoparticles	Thiolated xanthan gum	[95]
Triamcinolone acetonide	Hydrogel patch	κ-carrageenan, pectin	[72]
***Semi-solid drug dosage forms***			
Atorvastatin	Hydrogel	Chitosan, tragacanth gum, xanthan gum, low-methoxy amidated pectin, κ-carrageenan	[71]
Caspofungin	Hydrogel, paste, mouthwash,	Hyaluronic acid, chitosan, poloxamer 407	[96]
Fluconazole	Gel containing niosomes and solid lipid nanoparticles	Pluronic^®^ F127	[97]
anti-Candida peptide synthesized using a natural peptide of the hemolymph of crayfish *Procambarus clarkia*.	Hydrogel	Graft copolymer of xanthan gum and poly(N-isopropylacrylamide)	[98]
Fluconazole	Nanogel	Sulfhydryl-β-cyclodextrin, Carbopol 940, gelatin	[99]
Miconazole	Gel, film with solid lipid microparticles	Polyvinylpyrrolidone K90, hydroxyethy lcellulose	[100]
Fluconazole	Gel with nanotransfersomes	Hyaluronic acid	[101]
***Liquid drug dosage forms***			
Miconazole	Buccal microemulsion	Oleic acid, Tween 20, polyvinylpyrrolidone 400	[102]
Fluconazole	Film-forming spray	Hyaluronic acid, PVA, xanthan gum	[103]

## Data Availability

No new data were created or analyzed in this study. Data sharing is not applicable to this article.

## Abbreviations

The following abbreviations are used in this manuscript:

SI	Swelling Index (SI)
W_ad_	Work of Mucoadhesion
HPMC	Hydroxypropyl methylcellulose
PVA	Polivinyl Alcohol
RAS	Recurrent Aphthous Stomatitis
MAS	Magnesium Aluminum Silicate
HAL	Halloysite
LP-MN	Miconazole-loaded Liposomes
LP	Liposomes
FLU-SLNs	Fluconazole-loaded Solid Lipid Nanoparticles
ConBr	Lectin isolated from *Canavalia brasiliensis*
MaL	Lectin isolated from *Machaerium acutifolium*
κC	κ-carrageenan
λC	λ-carrageenan
SLS	Sodium Lauryl Sulfate
PECs	Polyelectrolyte Complexes
ATV/PG-Lip	Atorvastatin-loaded Propylene Glycol Liposomes
MIC	Minimum Inhibitory Concentration
BCS	Biopharmaceutics Classification System
SLNs	Solid Lipid Nanoparticles
SH-β-CD	Sulfhydryl-β-cyclodextrin
FL-NTFs	Fluconazole-loaded Nanotransfersomes
PEG 400	Polyvinylpyrrolidone 400
